# One- versus two-stage septic hip and knee revision surgery: a comparative cohort outcome study with short- to mid-term follow-up

**DOI:** 10.5194/jbji-10-185-2025

**Published:** 2025-06-02

**Authors:** Michelle M. J. Jacobs, Petra J. C. Heesterbeek, Karin Veerman, Jon H. M. Goosen

**Affiliations:** 1 Department of Orthopaedic Surgery, Sint Maartenskliniek, Hengstdal 3, 6574NA Ubbergen, the Netherlands; 2 Department of Research, Sint Maartenskliniek, Hengstdal 3, 6574NA Ubbergen, the Netherlands; 3 Department of Internal Medicine, Sint Maartenskliniek, Hengstdal 3, 6574NA Ubbergen, the Netherlands

## Abstract

**Introduction**: One-stage revisions seem to have similar reinfection rates compared to two-stage revisions for the treatment of periprosthetic joint infections based on retrospective cohort studies with a large variety of indications and treatment protocols. This study aimed to compare outcomes between comparable groups of one-stage and two-stage revision patients.

**Materials and methods**: We performed a retrospective cohort study, where equal numbers of one-stage and two-stage patients (knee: n=24 ; hip: n=40 ) were randomly included with the same inclusion and exclusion criteria. Patient characteristics and infection-related outcomes at latest follow-up were obtained via chart review. Functional outcomes (knee: Knee Society Score (KSS), range of motion (ROM), and visual analogue scale (VAS) pain and satisfaction; hip: Oxford Hip Score (OHS), Hip Disability and Osteoarthritis Outcome Score–Physical Function Shortform (HOOS-PS), VAS pain and satisfaction, and European Quality of Life 5 Dimensions 3 Level version (EQ5D-3L)) preoperatively (hip only) and at 1-year follow-up were extracted from a revision database. Outcomes were compared between one- and two-stage groups and for knee and hip cases separately.

**Results**: One- and two-stage groups were comparable for baseline characteristics. Reinfection occurred for both the knee and hip cohorts in one one-stage patient and one two-stage patient ( P=1.00 for both cohorts). More adverse events, of which two were spacer-related, were observed in two-stage hip patients ( n=7 ) compared to in one-stage patients ( n=2 ) ( P=0.13 ). Functional outcomes did not differ between one- and two-stage patients for both knee and hip cohorts.

**Conclusions**: This study showed no differences in terms of reinfection rates and functional outcomes between comparable groups of one- and two-stage septic knee and hip revision patients. A trend towards more adverse events in two-stage hip patients was seen, which was partly due to spacer complications.

## 1 Introduction

Periprosthetic joint infection (PJI) is a serious complication of knee and hip arthroplasties and occurs in approximately 1 % to 2 % of primary lower-limb joint replacements (Izakovicova et al., 2019). The golden standard for implant replacement is a two-stage revision, which has been extensively studied and has consistently shown low reinfection rates over the middle to long term. Goud et al. (2023) reviewed 46 two-stage hip revision studies and 48 two-stage knee revision studies and reported average reinfection rates of 8.4 % and 16.2%, respectively (Goud et al., 2023). Kunutsor et al. (2015, 2016) reported mid- to long-term reinfection rates of 7.9 % for two-stage hip revisions and 8.8 % for knee revisions (Kunutsor et al., 2015; Kunutsor et al., 2016).

Two-stage revisions require at least two surgical procedures and therefore a prolonged hospital stay and a period of immobilisation with partial weight bearing because of the spacer interval, resulting in substantial limitations in daily life activities (Vanhegan et al., 2012). They therefore impose a large burden on patients and on the healthcare system. An alternative to this procedure is a one-stage revision. The benefits are the need for only one surgery, less tissue damage, no spacer-related complications, lower healthcare costs, and potentially better functional outcomes owing to a shorter period of immobilisation (Haddad et al., 2015). One-stage revisions are most suitable for patients with preoperatively identified micro-organism(s) with known susceptibility to antibiotics, susceptibility to antibiotics with antibiofilm action, a good soft tissue envelope, and good bone stock (Rowan et al., 2018). Contraindications include failure of previous septic revision, immunosuppression, sepsis, and the presence of a sinus tract (Vanhegan et al., 2012; Rowan et al., 2018). The abovementioned meta-analyses reported reinfection rates of 5.7 % to 8.2 % after one-stage hip revisions and 7.6 % to 12.7 % after one-stage knee revisions, which did not differ from the two-stage revision reinfection rates in the middle to long term (Goud et al., 2023; Kunutsor et al., 2016; Kunutsor et al., 2015). While these results seem encouraging, it should be noted that these meta-analyses were biased due to a limited number of studies investigating the outcomes of one-stage revisions. In addition, only observational cohort studies were included, and the surgical (contra-)indications for one-stage revisions and the surgical protocols varied notably between studies. The body of evidence regarding whether one-stage revisions are a safe and beneficial option for hip and knee PJIs in selected patients is still limited due to these heterogeneities in existing literature.

This study set out to answer the following question: is there a difference in terms of reinfection rate between comparable groups (with respect to indication for surgery and patient characteristics) of one-stage and two-stage septic revision patients? We hypothesised that the reinfection rate does not differ between the two treatment options for both the hip and knee cohorts. In addition, we described and compared adverse events at latest follow-up and functional outcome scores and range of motion (ROM) after 1 year of follow-up between comparable one-stage and two-stage groups.

## 2 Methods

### 2.1 Study design and study population

**Figure 1:**
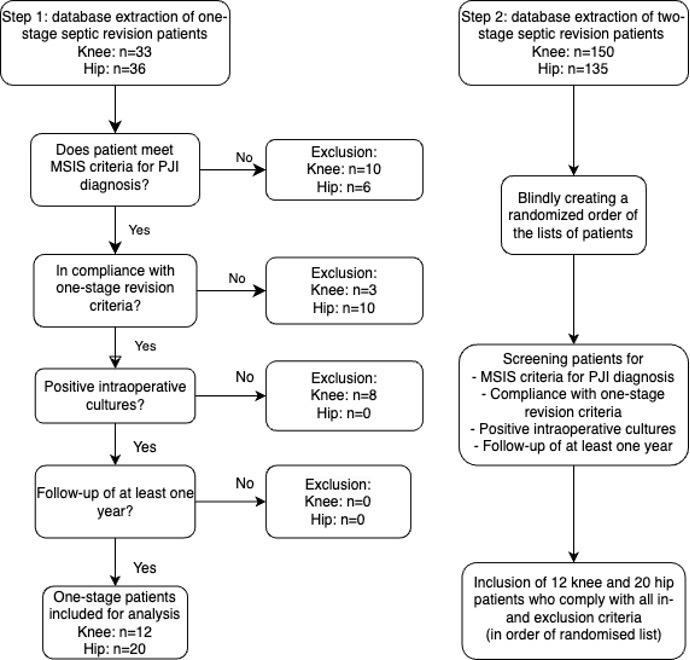
Flowchart of patient inclusion procedure.

This single-centre retrospective cohort study was conducted at the Sint Maartenskliniek in the Netherlands. Patients with a one-stage or two-stage septic hip or knee revision were selected from a prospective database consisting of data from patients who were operated on between 2013 and 2023. Hip and knee patients were investigated separately in this study. To create groups of two-stage patients that are comparable to one-stage patients, patients from both the one-stage and two-stage groups had to fulfil the criteria for one-stage septic revisions (Table 1). Shared decision making between the surgeon and patient was used to choose between a one-stage or two-stage procedure. Hence, we have a cohort of two-stage revision patients who do comply with the criteria for a one-stage septic revision. Patients were excluded from this study if they did not meet the MSIS (Musculoskeletal Infection Society) criteria for PJI (Parvizi et al., 2011), had negative intraoperative cultures, or had a follow-up duration of less than 1 year. A minimum of 1-year follow-up has been shown to be a reliable follow-up period for PJI treatment (Xu et al., 2020). One-stage patients were included first, and, subsequently, a random sample was taken from a large database of two-stage septic revision patients. Eligible two-stage patients were included in order of randomisation in a 1:1 ratio to one-stage patients (Fig. 1). All eligible patients were contacted and provided informed consent for the use of their medical data for this study.

**Table 1:** Criteria and contraindications for one-stage septic revision (Rowan et al., 2018; Thakrar et al., 2019).

Criteria	Contraindications
Pre-operatively identified micro-organism	Unidentified micro-organism
Known susceptibility to antibiotics	Difficult-to-treat micro-organism
Susceptibility to antibiotics with antibiofilm action	Presence of a sinus tract
Good soft tissue envelope	Failure of previous septic revision surgery
Good bone stock	Sepsis
	Immunodeficiency
	Peripheral vascular disease

### 2.2 Surgical procedures

All patients were treated with the same surgical protocols. Several experienced and fellowship-trained orthopaedic surgeons performed the procedures.

In all cases, necrotic and potentially infected tissue was thoroughly removed. For the knee, this occasionally included capsular and ligamentous tissue, necessitating the use of a hinged prosthesis for re-implantation. For the hip, we prioritised an endofemoral approach whenever possible to ensure the adequate removal of prosthetic material and bone cement. When this approach was insufficient, additional procedures, such as extended osteotomy, were performed. We consistently implanted a cemented dual-mobility cup due to the extensive removal of capsular and muscular tissue.

One-stage revisions consisted of first explanting the prosthesis, followed by obtaining six tissue cultures with clean, separate instruments. An extensive debridement and pulse lavage with 3 L of a betadine–0.9 % sodium chloride solution were performed. The wound was closed in two layers (fascia–capsule and skin). All antimicrobial drapes (including 3M Ioban antimicrobial incision foil), sterile gowns, and surgical instruments were replaced with new ones. After re-disinfection, the wound was reopened for re-implantation of the new prosthesis.

Two-stage revision patients underwent an explantation, where tissue cultures were obtained, and extensive debridement and pulse lavage were performed. Spacer implantation was a patient-specific choice and varied between patients in our study. Appropriate antimicrobial therapy between stages was discussed in the abovementioned multidisciplinary team. Re-implantation usually occurred 6 weeks after explantation without an antibiotic-free window. Again, new tissue cultures were obtained, and debridement was performed before the new prosthesis was implanted.

### 2.3 Microbiology and antimicrobial treatment

Preoperative cultures were obtained through joint fluid aspiration. During surgery, six tissue samples were collected for the culture. New sterile instruments were used for each sample.

Patients postoperatively received empirical antibiotic therapy with cefazolin until preliminary culture results became available. Patients were discussed in a multidisciplinary team, consisting of orthopaedic surgeons, infectious disease specialists, and microbiologists, where appropriate antimicrobial therapy was decided upon. One-stage revision patients received antibiotics for 12 weeks after revision, while two-stage revision patients received antibiotics for 6 weeks of after explantation and for 6 weeks after re-implantation. A 2-week antibiotic-free interval before re-implantation was used exclusively in patients without a spacer. If a patient with an antibiotic-free window had definitive negative cultures 2 weeks after re-implantation, antibiotic treatment was discontinued. In the case of streptococcal infections, rifampin was added. All antibiotic regimens can be found in Table S1 in the Supplement.

### 2.4 Data collection

Baseline data on patient characteristics (age, sex, body mass index (BMI), and co-morbidities) at the time of index revision and infection-related outcomes (reinfection rate, DAIR (debridement, antibiotic, and implant retention) incidence, all-cause revision, adverse events, and mortality) at the latest recorded clinical follow-up were obtained via chart review. For knee functional outcomes, we used the functional, clinical, and total scores from the Knee Society Score (KSS), range of motion (ROM), visual analogue scale (VAS) pain scores, and VAS satisfaction scores at 1-year follow-up only as the preoperative data were too incomplete to be used for analyses. We used the ROM scores from the KSS, where one point represents 5 degrees of motion. The scores were multiplied by 5 to get an approximation of the total range of motion in degrees. The clinical and functional KSS and VAS scores run on a scale from 0 to 100, and the total KSS score runs on a scale from 0 to 200, with the latter being the best outcome. Functional outcomes for hip patients include the Oxford Hip Score (OHS, scale of 14–70; lower is a better outcome), Hip Disability and Osteoarthritis Outcome Score–Physical Function Shortform (HOOS-PS, scale of 0–100; lower is a better outcome), VAS pain scores for rest and activities (scale of 0–100; lower is a better outcome), VAS satisfaction scores (scale of 0–100; higher is a better outcome), and European Quality of Life 5 Dimensions 3 Level version (EQ5D-3L; higher is a better outcome). Functional hip outcomes were recorded preoperatively and at 1-year follow-up.

### 2.5 Statistical analyses

Statistical analyses were performed using IBM SPSS Statistics version 29.0.1.0 and were done for hip and knee patients separately. All data were tested for normality with the Shapiro–Wilk test. Descriptive analysis was performed for baseline patient characteristics, microbiology data, and functional outcomes. Data are presented as the mean ( ± standard deviation, SD) in the case of normally distributed data or the median (interquartile range, IQR) in the case of nonparametric data. Differences in reinfection rates, all-cause revisions, adverse events, and mortality at latest follow-up were tested with Chi-squared or Fisher's exact test. Reinfection was defined following the Delphi criteria (Diaz-Ledezma et al., 2013):

1. infection eradication, characterised by a healed wound without fistula, drainage, or pain, and no infection recurrence caused by the same organism strain;
2. no subsequent surgical intervention for infection after re-implantation surgery;
3. no occurrence of PJI-related mortality.

Knee functional outcomes were compared between one-stage and two-stage groups at 1-year follow-up with independent-sample t tests or Wilcoxon–Mann–Whitney tests in the case of nonparametric data. In the case of missing data, the 3-month follow-up data were used as a substitute as van Kempen et al. (2013) showed that 3-month postoperative scores are suggestive of later outcomes (van Kempen et al., 2013). For hip functional outcomes, delta scores were calculated by subtracting the preoperative scores from the scores at 1-year follow-up. Independent-sample t tests or Wilcoxon–Mann–Whitney tests were used to test for differences in delta scores between one-stage and two-stage groups. The VAS satisfaction score was not recorded preoperatively; hence, only the scores at 1-year follow-up were compared between groups. Statistical significance was set at P≤0.05 .

## 3 Results

### 3.1 Patient selection and baseline characteristics

After screening for inclusion and exclusion criteria, 24 knee patients and 40 hip patients were included (Fig. 1). Patient characteristics are displayed in Table 2 and were comparable between one-stage and two-stage groups. Mono-bacterial infections were equally common in one-stage and two-stage patients. Three patients had a discrepancy between preoperative and intraoperative cultures. In all three cases, *Cutibacterium acnes* was also found in intraoperative cultures. A detailed description of microbiology data can be found in Table S2. Regarding infection-related outcomes and adverse events, the mean follow-up duration for knee patients was 40 (SD = 24) months for the one-stage group and 65 (SD = 14) months for the two-stage group. Hip patients had a mean follow-up duration of 62 (SD = 32) months for one-stage patients and 82 (SD = 28) months for two-stage patients.

**Table 2:** Characteristics of the patients included in this study ( n= 64).

	Knee				Hip			
	Total ( n=24 )	One stage ( n= 12)	Two stage ( n= 12)	P value^*^	Total ( n= 40)	One stage ( n= 20)	Two stage ( n= 20)	P value^*^
Sex, males	11	6	5	1.00	23	13	10	0.34
Age, years	66 (9)	69 (9)	63 (9)	0.08	67 (11)	68 (13)	67 (9)	0.83
BMI, kg m^−2^	28.6 (5.6)	28.9 (5.4)	28.3 (6.0)	0.78	26.7 (23.7; 31.2)	25.8 (24.4; 30.9)	27.7 (23.0; 27.7)	0.84
Co-morbidity								
– Diabetes mellitus	4	4	0	0.09	8	4	4	1.00
– Auto-immune disease	0	0	0	n/a	3	2	1	1.00
– Immunosuppressants	2	2	0	0.48	2	1	1	1.00
Mono-bacterial PJI	18	7	11	0.16	39	19	18	1.00
Culture discrepancy	2	1	1	1.00	1	1	0	1.00

N denotes number, SD denotes standard deviation, and BMI denotes body mass index. Data are displayed as the number, mean ( ± SD), or median (IQR). ^*^ Two-sided independent-sample t test or Wilcoxon–Mann–Whitney test. For binary variables, the Chi-squared test was used if the count ≥ 5, and the two-sided Fisher's exact test was used if count < 5 for nominal variables. Statistical significance is set at P≤ 0.05. n/a: not applicable

### 3.2 Knee infection-related outcomes

Reinfection occurred in two patients, with one patient from the one-stage group and one from the two-stage group (Table 3; P=1.00 ). A description of all reinfection and revision patients can be found in Table S3. One patient from the one-stage group had recurring patellar luxations, for which the patient underwent an additional tibial tuberosity osteotomy. This was scored as an adverse event.

**Table 3:** Infection-related outcomes of knee and hip one- and two-stage septic revisions.

	Knee				Hip			
	Total	One stage	Two stage	P	Total	One stage	Two stage	P
	( n= 24)	( n= 12)	( n= 12)	value^*^	( n= 40)	( n= 20)	( n= 20)	value^*^
Reinfection	2	1	1	1.00	2	1	1	1.00
Revision	2	1	1	1.00	3	1	2	1.00
DAIR	2	0	2	0.48	1	0	1	1.00
Adverse events	1	1	0	1.00	9	2	7	0.13
Mortality	0	0	0	n/a	2	1	1	1.00

Data are presented as a number. DAIR denotes debridement, antibiotic, and implant retention, and n/a denotes not applicable. ^*^ The Chi-squared test was used if the count was ≥ 5, and the two-sided Fisher's exact test was used if count < 5. Statistical significance is set at P≤ 0.05.

### 3.3 Hip infection-related outcomes

Reinfection occurred in two patients, with one patient from the one-stage group and one from the two-stage group (Table 3; P=1.00 ). Although not statistically significantly different ( P=0.13 ), we observed more adverse events in two-stage patients. Adverse events in the one-stage group included femoral nerve axonotmesis ( n=1 ) and a periprosthetic fracture ( n=1 ). In the two-stage group, two procedure-related adverse events were associated with the use of a spacer, namely a trochanter major fracture during spacer use ( n=1 ) and lower-extremity deep venous thrombosis (DVT) during spacer immobilisation ( n=1 ). Other adverse events included de novo atrial fibrillation ( n=1 ), a trochanter major fracture during uncemented stem removal ( n=1 ), superinfection after re-implantation ( n=2 ), and upper-extremity DVT related to PICC (peripherally inserted central catheter) line ( n=1 ).

### 3.4 Functional outcomes

The functional outcomes of knee patients are displayed in Table 4a. At 1-year follow-up, functional outcomes were comparable between one-stage and two-stage patients. A descriptive analysis of the functional outcomes of hip patients is displayed in Table 4b. Delta scores of functional outcomes between groups were not statistically different. Moreover, the VAS satisfaction score at 1-year follow-up was comparable between the groups.

**Table 4:** Functional outcomes.

**(a)** Functional outcomes of knee patients at 1-year follow-up.			
	One stage	Two stage	P value^*^
Clinical knee score	79.8 (15.9)	82.0 (13.8)	0.75
Functional knee score	70.0 (20.6)	68.5 (18.9)	0.87
Total knee score (KSS)	149.8 (29.1)	150.5 (25.3)	0.96
ROM	120 (104; 124)	125 (120–125)	0.20
VAS pain	23.5 (10.5; 47.3)	20.0 (12.0; 54.0)	0.93
VAS satisfaction	79.5 (62.3; 97.8)	82.0 (76.0; 96.0)	0.83
KSS denotes Knee Society Score, ROM denotes range of motion, and VAS denotes visual analogue scale.			
Data are displayed as the mean ( ± SD) or median (IQR). ^*^ Two-sided independent-sample t test or			
Wilcoxon–Mann–Whitney test. Statistical significance is set at P≤ 0.05.			

**(b)** Functional outcomes of hip patients at different time points.								
	One stage			Two stage			P value^*^	
	Preop	1 year	Delta score	Preop	1 year	Delta score		
OHS	43.2 (10.7)	19.8 (7.4)	- 14.5 (11.1)	37.7 (11.2)	23.0 (15.0; 37.0)	- 12.8 (9.7)	0.64	
HOOS-PS	50.2 (22.6)	24.6 (17.9)	- 25.8 (19.0)	60.3 (31.6)	34.7 (27.8)	- 27.3 (31.5)	0.88	
VAS pain rest	20.9 (18.2)	4.3 (6.0)	- 12.5 ( - 23.5; - 2.8)	44.9 (27.9)	6.0 (0.0; 27.0)	- 24.0 ( - 45.0; - 12.0)	0.13	
VAS pain active	55.9 (28.8)	19.8 (22.0)	- 36.1 (31.8)	65.9 (16.9)	22.0 (4.0; 58.0)	- 38.4 (28.3)	0.82	
VAS satisfaction	–	75.1 (24.1)	–	–	79.5 (26.8)	–	0.32	
EQ5D-3L	0.67 (0.25; 0.78)	0.78 (0.75; 0.90)	0.16 (0.01; 0.57)	0.42 (0.31)	0.64 (0.32)	0.23 (0.00; 0.52)	0.87	

Preop denotes preoperative, OHS denotes Oxford Hip Score, HOOS-PS denotes Hip Disability and Osteoarthritis Outcome Score–Physical Function Shortform, and EQ5D-3L denotes European Quality of Life 5 Dimensions 3 Level version. Data are presented as the mean ( ± SD) for normally distributed data or the median (IQR). ^*^ Two-sided independent-sample t test or Wilcoxon–Mann–Whitney test. Statistical significance is set at P≤ 0.05.

## 4 Discussion

This study compared the mid-term outcomes of one-stage and two-stage septic revisions between comparable groups of patients, thoroughly selected in terms of surgical indications and patient characteristics. To the best of our knowledge, this study is the first to investigate these outcomes between comparable groups of one-stage and two-stage patients for hip and knee cohorts separately. Similar numbers of patients with reinfection and re-revision were found in both hip and knee cohorts. Furthermore, we described functional outcomes at 1-year follow-up and found no difference between one- and two-stage patients.

Reinfection occurred in two knee patients and in two hip patients. Although the number of included patients is small, our finding that reinfection does not differ between one-stage and two-stage groups is in line with recent large observational studies and meta-analyses comparing reinfection rates of one-stage and two-stage revisions (Goud et al., 2023; Kunutsor et al., 2015; Kunutsor et al., 2016; Masters et al., 2013; Matar et al., 2021). The current available literature about the safety of one-stage septic revisions is mostly from observational studies, where the groups of patients treated with either a one- or two-stage procedure are difficult to compare since it is often not clear how they were selected or due to the fact that they were selected differently for both procedures. However, the finding that one-stage septic revisions in selected patients have similar reinfection rates compared to two-stage revisions has been repeatedly reproduced, and our study adds to that by again showing no difference in reinfection between one- and two-stage patients.

Three patients (one one-stage knee, one one-stage hip, and one two-stage knee) had a discrepancy between preoperative and intraoperative culture results. All three patients had an additional *C. acnes* in the intraoperative cultures. The clinical relevance of this finding is uncertain. Detecting an additional micro-organism indicates a polymicrobial infection that was not identified preoperatively. Since polymicrobial infections can contraindicate one-stage septic revisions (Haddad et al., 2015), such patients might have been inappropriately treated with this approach. Notably, a one-stage knee patient with an undetected polymicrobial infection experienced reinfection. While it is unclear if this failure resulted from the polymicrobial infection, the case highlights the importance of accurate preoperative cultures as misidentifying causative micro-organisms can lead to suboptimal treatment.

The occurrence of adverse events was comparable between one- and two-stage groups. We did observe slightly more adverse events for two-stage hip patients ( n=7 ) compared to the one-stage patients ( n=2 ), but this result was not statistically significant ( P=0.13 ). Interestingly, two patients from the two-stage group had a procedure-related adverse event, namely a trochanteric fracture during spacer use and DVT during spacer immobilisation. It is important to note that spacer-related complications occur exclusively in two-stage revision patients, which explains why these patients inherently have a higher risk of complications. A study by Thiesen et al. (2021) compared the short-term complication rate between one-stage and two-stage hip and knee revisions with large groups of comparable patients (Thiesen et al., 2021). They found no difference in surgery-related complications (such as DVT or nerve injury), which is in line with the findings from the present study. However, they did describe a statistically significant higher occurrence of medical complications, e.g. atrial fibrillation and pneumonia, in two-stage patients. One-stage patients might therefore have a net lower risk of treatment-related complications, which is another argument for considering this treatment option for eligible patients. In the future, larger comparative studies should also focus on these procedure-related adverse events.

With respect to knee functional outcomes, all five parameters were comparable between one- and two-stage groups. This indicates that patients function at a similar level 1 year after revision regardless of whether they had a one- or two-stage procedure. The satisfaction scores were very similar to each other. Very few studies have investigated this subject. Budin et al. (2022) also found no difference in terms of patient-reported outcome measures (PROMs) between one-stage and two-stage septic knee revision patients with an even longer mean follow-up period of 54.5 months. For hip patients, we described improvements in all preoperative scores at 1-year follow-up. There was a notably larger improvement in VAS resting pain score in the two-stage group (median improvement of 24 points) compared to in the one-stage patients (median improvement of 12.5 points), but this result and the other functional outcomes did not differ significantly. Previous reports did find clinically relevant higher PROMs in one-stage revision patients compared to propensity-score-matched two-stage patients at an average follow-up time of 16.7 months (Tirumala et al., 2021). Perhaps there might be a difference in terms of functional outcomes, but we were unable to replicate this result statistically due to our lower numbers.

One strength of the present study is that it describes both infection-related and functional outcomes of one- and two-stage groups that are comparable in terms of surgery indications and baseline patient characteristics, and all patients were treated with a standardised surgical protocol. The comparisons are therefore more reliable than comparing unrelated groups of one- and two-stage patients. Another strength is that data on functional outcomes were collected prospectively, thus eliminating recall bias. A limitation of this study is the small number of patients per group, which narrowed our options for statistical analyses and firm conclusions. Another limitation is that it remains unclear as to why many patients who fulfilled the one-stage criteria underwent a two-stage procedure. This might be partially explained by the increasing worldwide awareness of the strengths of one-stage revisions in the last decade that has led to a wider implementation of one-stage septic revisions, including in our hospital. Other external factors, such as surgeon preference or patient request, might also have contributed. It was not possible to investigate and describe these factors as they were not recorded. By using a randomised selection of the two-stage patients, we attempted to mitigate this limitation. Despite these limitations, we still believe that this study is a valuable addition to the current body of literature comparing the outcomes of one- and two-stage septic revisions due to the careful design and comparable groups and the detailed description of patients with a reinfection or revision. The current study design can provide useful data, and we encourage this type of study or randomised prospective studies, such as the INFORM trial (Strange et al., 2016), to be undertaken at a larger scale.

## 5 Conclusions

In conclusion, this study investigated mid-term outcomes between comparable one-stage and two-stage proven-septic knee and hip revision arthroplasty cases. Patients were thoroughly selected based on infection parameters and traditional one-stage-revision surgical indications. No differences in terms of the incidence of reinfection all-cause re-revisions were found, which is in line with previous studies. A trend towards more adverse events in two-stage hip patients compared to in one-stage patients was seen, which was partly due to procedure-related spacer complications. Functional outcomes at 1-year follow-up were similar between one- and two-stage knee patients, while one- and two-stage hip patients improved at similar rates from their preoperative condition to 1-year follow-up.

## Supplement

10.5194/jbji-10-185-2025-supplement The supplement related to this article is available online at https://doi.org/10.5194/jbji-10-185-2025-supplement.

## Supplement

10.5194/jbji-10-185-2025-supplement
10.5194/jbji-10-185-2025-supplement The supplement related to this article is available online at https://doi.org/10.5194/jbji-10-185-2025-supplement.

## Data Availability

The data presented in this study are available from the corresponding author upon reasonable request.

## Appendix

The supplement related to this article is available online at https://doi.org/10.5194/jbji-10-185-2025-supplement.
