# Supplementary material for: One- versus two-stage septic hip and knee revision surgery: a comparative cohort outcome study with short- to mid-term follow-up
Source: J Bone Jt Infect. 2025 Jun 2;10(3):185–92. doi: 10.5194/jbji-10-185-2025 (PMC12163721; doi:10.5194/jbji-10-185-2025)
Supplement: The supplement related to this article is available online at https://doi.org/10.5194/jbji-10-185-2025-supplement. [file jbji-10-185-2025-supplement.pdf]

Supplement of J. Bone Joint Infect., 10, 185–192, 2025
https://doi.org/10.5194/jbji-10-185-2025-supplement
© Author(s) 2025. CC BY 4.0 License.

Supplement of

One- versus two-stage septic hip and knee revision surgery: a comparative cohort outcome study with short- to mid-term follow-up

Michelle M. J. Jacobs et al.

Correspondence to: Michelle M. J. Jacobs (mmjjacobs2@gmail.com)

The copyright of individual parts of the supplement might differ from the article licence.

Table S1. Causative micro-organism and antimicrobial regiment for all patients (n=64).

Knee patients				Hip patients			
One stage	Micro-organism	Antibiotic	Rifampin	One stage	Micro-organism	Antibiotic	Rifampin
1	Staphylococcus spp	Ciprofloxacin	Yes	1	S. epidermidis	Ciprofloxacin	Yes
2	S.warneri<br>Staphylococcus spp.	Teicoplanin	No, due to allergy	2	S. haemolyticus	Teicoplanin	No, due to allergy
3	S. epidermidis	Ciprofloxacin	Yes	3	S. caprae	Clindamycin	Yes
4	S. epidermidis,<br>S capitis,<br>C. acnes	Clindamycin	Yes	4	S. milleri<br>C. acnes	Ciprofloxacin	Yes
5	S. epidermidis	Ciprofloxacin	Yes	5	S. epidermidis	Ciprofloxacin	Yes
6	S. epidermidis<br>C. acnes	Ciprofloxacin	Yes	6	S. aureus	Ciprofloxacin	Yes
7	S. epidermidis,<br>C. acnes	Clindamycin	No, due to allergy	7	C. acnes	Clindamycin	No
8	S. epidermidis,<br>C. acnes	Clindamycin	Yes	8	S. caprae	Ciprofloxacin	Yes
9	Streptococcus spp.	Clindamycin	Yes	9	S. capitis	Ciprofloxacin	Yes
10	S. epidermidis	Clindamycin	Yes	10	S. capitis	Ciprofloxacin	Yes
11	C. acnes	Clindamycin	No	11	S. aureus	Ciprofloxacin	Yes
12	S. caprae	Ciprofloxacin	Yes	12	Streptococcus spp	Clindamycin	No
Two stage				13	S. caprae	Ciprofloxacin	Yes
1	C. acnes	Clindamycin	No	14	C. acnes	Clindamycin	No
2	S. milleri	Amoxicillin	No	15	S. aureus	Clindamycin	Yes
3	S. capitis<br>C. acnes	Ciprofloxacin	Yes	16	Streptococcus spp.	Linezolid	No
4	S. epidermidis	Ciprofloxacin	Yes	17	E. coli	Amoxicillin	No
5	S. epidermidis	Ciprofloxacin	Yes	18	S. epidermidis	Ciprofloxacin	Yes
6	Streptococcus spp.	Amoxicillin	No	19	S. vestibularis	Clindamycin	No
7	Granulicatella<br>adiacens	Clindamycin	No	20	S. epidermidis	Clindamycin	Yes
8	S. caprae	Ciprofloxacin	Yes	Two stage			
9	Corynebacterium spp	Linezolid	No	1	C. acnes	Amoxicillin	No
10	C. acnes	Clindamycin	No	2	S. epidermidis	Ciprofloxacin	Yes

11	S. aureus	Clindamycin	Yes	3	S. aureus	Clindamycin	Yes
12	C. acnes	Clindamycin	No	4	S. aureus	Clindamycin	Yes
				5	S. aureus	Ciprofloxacin	Yes
				6	S. aureus	Clindamycin	Yes
				7	S. aureus	Ciprofloxacin	Yes
				8	S. epidermidis<br>Enterobacter aerogenes	Ciprofloxacin<br>Teicoplanin	Yes
				9	C. acnes<br>S. epidermidis	Ciprofloxacin<br>Vancomycin	Yes
				10	S. capitis	Ciprofloxacin	Yes
				11	S. aureus	Ciprofloxacin	Yes
				12	S. epidermidis	Ciprofloxacin	Yes
				13	S. epidermidis	Clindamycin	Yes
				14	S. aureus	Ciprofloxacin	Yes
				15	Staphylococcus spp.	Ciprofloxacin	Yes
				16	S. lugdunensis	Ciprofloxacin	Yes
				17	C. acnes	Clindamycin	No
				18	S. capitis	Moxifloxacin	Yes
				19	Enterococcus faecium	Teicoplanin	No
				20	C. acnes	Clindamycin	No

Table S2. Cultured micro-organisms (n=71) from all included patients (n=64)

	Knee			Hip		
	Total	1st	2st	Total	1st	2st
Monobacterial PJI, n	18	7	11	39	19	18
Micro-organism, n	30	17	13	41	21	20
Species, n						
- Staphylococcus spp	15	10	5	28	13	15
-S. aureus	1	0	1	10	3	7
-S. epidermidis	9	7	2	9	4	5
-S. lugdunensis	0			1	0	1
-S. caprae	2	1	1	3	3	0
-S. capitis	1	0	1	4	2	2
-S. haemolyticus	0			1	1	0
- Streptococcus spp	4	2	2	4	2	0
-S. milleri	1	0	1	1	1	0
-S. warneri	1	1	0	0		
-S. vestibularis	0			1	1	0
- Cutibacterium acnes	9	5	4	7	3	4
- Corynebacterium spp	1	0	1	0		
- Anaerobes	1	0	1	0		
- Enterococcus spp	0			1	0	1
- Enterobacter spp	0			1	0	1
- Escherichia coli	0			1	1	0

N, number; 1st, one-stage; 2st, two-stage

Table S3. Description of patients with reinfection, revision or DAIR.

Case	Inclusion group	Reinfection/revision/DAIR	Time to failure (months)	Cultures PJI	Cultures new revision/DAIR	Description
1	One-stage knee	Reinfection with two-stage revision	19	*S. epidermidis* *Cutibacterium acnes*	*S. epidermidis*	Fifteen months after revision, the patellar component was removed due to loosening. Intra-operative cultures were negative. However, patient had persisting diffuse joint effusion and erythema. Two separate intra-articular aspirations showed a resistant strain of *S. epidermidis.* Patient was treated with a two-stage revision and antibiotics. He recovered well and showed no signs of infection at latest follow-up (+2 years after two-stage revision).
2	Two-stage knee	Revision (not infection related)	50	*Cutibacterium acnes*	Negative	At fifty months follow-up patient had a revision due to asesptic loosening of the tibial component. Intraoperative cultures were negative. Patient is doing well and has shown no signs of infection.
3	Two-stage knee	Reinfection with DAIR	<1	*Granulicatella adiacens*	*S. epidermidis*	Patient had persisting wound drainage during three weeks after reimplantation. A DAIR was performed. Cultures showed a new micro-organism. Patient was treated with teicoplanin and rifampicin for twelve weeks and recovered fully.
4	Two-stage knee	New infection with DAIR (unrelated to original PJI)	49	*Cutibacterium acnes*	Negative	Patient has history of venous insufficiency and suffered from a medial ulcer at the ipsilateral ankle. He then developed a cellulitis originating from the ulcer, which spread to this TKA one week after onset of symptoms, despite antibiotic treatment. Patient was treated with a DAIR, which revealed much intra-articular pus. Intraoperative cultures were negative, likely due to prior antibiotic treatment for ten days. Patient was treated with clindamycin for 12 weeks and recovered well.

5	One-stage hip	Reinfection and revision (the latter not infection related)	3	*E. coli*	Not available	Patient developed an intraabdominal abscess with the same micro-organism as the PJI, due to insufficient infection control after revision. He receives chronic suppressive antimicrobial therapy with amoxicillin, which he tolerates well. Additionally, patient fell three months after the one-stage revision and sustained a periprosthetic fracture (Vancouver B2). A femoral stem revision and fracture fixation were performed.
6	Two-stage hip	Revision (not infection related)	99	*S. aureus*	Negative	Ninety-nine months after revision, patient presented to the clinic with complaints of pain while mobilizing. Imaging showed radiolucent lines around the acetabular cup. A cup revision was performed. Intra-operative cultures were negative. Patient recovered well.
**7**	Two-stage hip	Revision (not infection related) with unexpectedly positive cultures with new micro-organism	76	*S. aureus*	*S. capitis*	Patient presented to the clinic with hip pain. Imaging showed a migrated loose cup. Preoperative joint aspiration was negative, but intra-operative cultures were unexpectedly positive. Patient was treated with clindamycin and rifampicin for twelve weeks. She showed no signs of infection at latest follow-up and a new joint aspiration was negative.
8	Two-stage hip	Reinfection and repeated DAIR	<1	*Cutibacterium acnes*	DAIR 1: *S. epidermidis* *Citrobacter koseri*<br><br>DAIR 2: *S. epidermidis* *Cutibacterium acnes*	Patient had persistent wound drainage and elevated serum inflammation markers, for which he was treated with two DAIRs within one month after reimplantation. Intraoperative cultures showed a superinfection. Patient was treated with vancomycin, ciprofloxacin and rifampicin and he recovered well.
